# Textured insoles may improve some gross motor balance measures but not endurance measures in children with motor coordination issues. A randomised controlled feasibility trial

**DOI:** 10.1002/jfa2.12036

**Published:** 2024-07-01

**Authors:** Helen A. Banwell, Margarita Tsiros, Jessica Coventry, Narelle Ryan, Cylie M. Williams

**Affiliations:** ^1^ Allied Health and Human Performance University of South Australia Adelaide South Australia Australia; ^2^ Innovation, Implementation and Clinical Translation in Health (IIMPACT) University of South Australia Adelaide South Australia Australia; ^3^ Alliance for Research in Exercise Nutrition and Activity University of South Australia Adelaide South Australia Australia; ^4^ Monash University School of Primary and Allied Health Frankston Victoria Australia

**Keywords:** balance, developmental coordination disorder, endurance, feasibility, footwear, motor coordination, orthoses, paediatrics, physiotherapy, podiatry

## Abstract

**Background:**

Motor coordination concerns are estimated to affect 5%–6% of school‐aged children. Motor coordination concerns have variable impact on children's lives, with gait and balance often affected. Textured insoles have demonstrated positive impact on balance and gait in adults with motor coordination disorders related to disease or the ageing process. The efficacy of textured insoles in children is unknown. Our primary aim was to identify the feasibility of conducting a randomised controlled trial involving children with motor control issues. The secondary aim was to identify the limited efficacy of textured insoles on gross motor assessment balance domains and endurance in children with movement difficulties.

**Methods:**

An assessor‐blinded, randomised feasibility study. We advertised for children between the ages of 5–12 years, with an existing diagnosis or developmental coordination disorder or gross motor skill levels assessed as 15th percentile or below on a norm‐referenced, reliable and validated scale across two cities within Australia. We randomly allocated children to shoes only or shoes and textured insoles. We collected data across six feasibility domains; demand (recruitment), acceptability (via interview) implementation (adherence), practicality (via interview and adverse events), adaptation (via interview) and limited efficacy testing (6‐min walk test and balance domain of Movement ABC‐2 at baseline and 4 weeks).

**Results:**

There were 15 children randomised into two groups (eight received shoes alone, seven received shoes and textured insoles). We experienced moderate demand, with 46 potential participants. The insoles were acceptable, however, some parents reported footwear fixture issues requiring modification. The 6‐min walk test was described as problematic for children, despite all but one child completing. Social factors impacted adherence and footwear wear time in both groups. Families reported appointment locations and parking impacting practicality. Underpowered, non‐significant small to moderate effect sizes were observed for different outcome measures. Improvement in balance measures favoured the shoe and insole group, while gait velocity increase favoured the shoe only group.

**Conclusion:**

Our research indicates that this trial design is feasible with modifications such as recruiting with a larger multi‐disciplinary organisation, providing velcro shoe fixtures and using a shorter timed walk test. Furthermore, progressing to a larger well‐powered randomised control trial is justified considering our preliminary, albeit underpowered, efficacy findings.

**Trial Registration:**

This trial was retrospectively registered with the Australian and New Zealand Clinical Trial Registration: ACTRN12624000160538.

**Abbreviations**
6MWT: 6‐min walk test
DCD: developmental coordination disorder
EOI: expression of interest
NR: not recorded
SD: standard deviation

## 1 BACKGROUND

Motor coordination disorders, such as developmental coordination disorder (DCD), are estimated to affect 5%–6% of school‐aged children [1]. Children with motor coordination disorders experience increased variability and inefficiencies in gait patterns, impaired ball skills, poor handwriting and other fine and gross motor skills disruption. These disruptions often result in extensive impacts on activities of daily life [2, 3]. Reductions in gross motor skills are also a key predictor of a child's lack of engagement with physical activity [4]. Consequently, children with motor coordination issues are more likely to experience overweight or obesity, have reduced physical fitness participation, lower fitness outcomes and report lower quality of life [4, 5]. Young people with motor coordination disorders also have broader mental health impacts, with a higher prevalence of anxiety and depression and more frequent episodes of frustration, aggression and suicide compared to their typically developing peers [5, 6].

Children with motor coordination disorders benefit from multi‐faceted management approaches. Intervention techniques frequently relate to tasks or processes associated with activities of daily living [3, 4, 6, 7]. Gait changes are often key to these impacts, with children with motor coordination disorders also commonly displaying low muscle tone and fatigue, ligamentous laxity, toe walking, excessive tripping, impaired proprioception and complaining of musculoskeletal pain [1]. Our previous research identified that children with motor coordination disorders display significantly slower gait speed, endurance and have more gait variability compared to children without these concerns [2]. This finding gives rise to consideration of how interventions known to change gait may impact children with motor coordination disorders.

One intervention that is novel in the adult population is textured insoles. Textured insoles are purported to improve sensorimotor control, with a positive impact observed in both multi‐directional postural sway in healthy young adults [8], community‐dwelling older adults [9] and adults with Parkinson's disease [10, 11]. Textured insoles may therefore be a viable option to improve gait or gross motor skills or endurance in people with known motor control issues, especially in childhood.

Given the gap in evidence, no known use of this intervention for children with motor coordination disorders and unknown recruitment ability, a feasibility trial is an appropriate exploration to identify if an adequately powered randomised trial might be achievable. A feasibility trial, described as ‘a study that can help investigators prepare for full‐scale research’ [12] allows investigators to identify enablers for engagement in research and make recommendations for protocol modifications, particularly around recruitment [13, 14, 15]. Therefore, our primary aim was to identify the feasibility of conducting an appropriately powered, multi‐site randomised controlled trial involving children with motor control issues. The secondary aim was to explore calculations to identify if there is a trend or suggestion of impact from the intervention and the suitability of our outcome measures for detecting change within this population [12].

## 2 METHODS

This study is reported in accordance with the Consolidated Standards of Reporting Trials (CONSORT) extension relating to pilot and feasibility trials [16]. Ethical approval for this project was obtained by the Human Research Ethics Committee of the University of South Australia (Approval number 204442). We obtained written, informed consent from parents/caregivers prior to data collection and all participants gave assent to be involved in all tasks across all sessions. This trial was retrospectively registered with the Australian and New Zealand Clinical Trial Registration: ACTRN12624000160538.

### 2.1 Study design

This study was an assessor‐blinded, parallel‐group randomised control trial using a mixed‐method design to capture qualitative and quantitative outcomes. Study feasibility was based on collection of outcome measures across six of the eight domains of Bowen's framework for feasibility studies seeking *Demand*, *Acceptability*, *Implementation*, *Practicality*, *Adaptation* and/or *Limited efficacy* testing of the trial processes and intervention [12].

### 2.2 Study setting

Recruitment and data collection occurred at two sites; a health and medical service attached to a University in South Australia [University] and a private podiatry service in metropolitan Victoria (Australia) [Private practice]. In consideration of the different client base and clinician capacity between sites, the University aimed to enrol 16 participants, with the private practice aiming for 4 participants. The University site is a dedicated health and medical clinic, co‐located at the City West campus of the University of South Australia (North Tce, Adelaide) that offers paediatric allied health services to the community. The Private Practice (Cheltenham, Victoria) was a general podiatry clinic staffed by three podiatrists with 2 days of dedicated paediatric service delivery.

### 2.3 Participant eligibility and recruitment

Children were eligible to participate in this study if they:

- were aged 5–12 years, and
- had not worn foot or leg orthoses in the past 6 months, and
- had an existing diagnosis of DCD or had been assessed on the 15th percentile or below for gross motor skills using a norm‐referenced validated and reliable gross motor assessment battery (e.g., Movement Assessment Battery for Children, 2nd Edition [Movement ABC‐2] [17, 18]). This was in consideration that children scoring at the 15th percentile or below have or are considered at risk of having significant movement difficulty [19].

Participants were excluded if they had a disorder or condition that impacted their ability to participate in gross motor assessment or walk for 6 minutes or more unaided, including significant joint hypermobility that had previously resulted in joint subluxation and/or dislocation, or were unable to verbally communicate with the researchers. All parents or carers provided written consent and children assented prior to participation.

The a priori recruitment target was 20 participants. Recruitment occurred over 6 months, involving passive and active phases. Passive recruitment involved flyer advertising with the recruitment sites and via the authorship groups' personal social media accounts, running for 12 weeks. Active recruitment was instigated in week 13 of the recruitment period involving two paid Facebook™ adverts targeting parents and grandparents of children aged between 5 and 12 years across metropolitan South Australia and Victoria. Each advert ran for 2 weeks.

### 2.4 Randomisation of interventions

Participants were randomly allocated into a control group [shoe group] or intervention group [shoe and insole group]. Randomisation was conducted using a computer‐generated random number sequencer, completed a priori as batches of six in permuted blocks, in consideration of potential recruitment concerns [20]. Randomisation was conducted by an author not involved in screening or assessing participants (HB), held on a password‐protected excel file only accessible to the two authors dispensing the intervention (HB and CW).

### 2.5 Interventions

**FIGURE 1:**
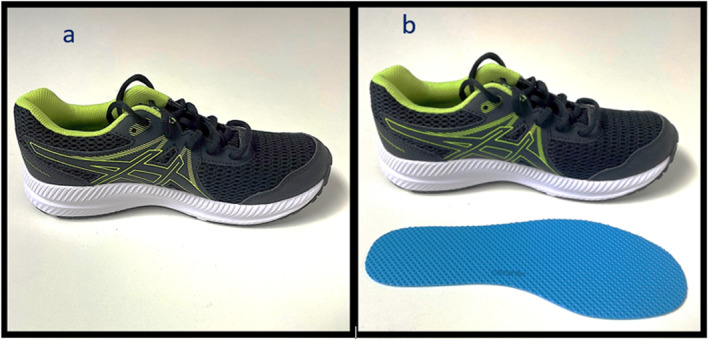
The ASICS contend sneakers (a) and Naboso activation textured insoles (b) used within this study.

The shoe group received standardised shoes (ASICS Contend sneakers^TM^) and the intervention group received standardised shoes (ASICS Contend sneakers^TM^) with a Naboso Activation insole^TM^ fitted (Figures 1a,b). The footwear was an athletic shoe including lacing fixtures, firm heel counter and semi‐flexible sole. Those in the intervention group were also asked to wear thin socks as per the textured insole manufacturer's recommendations.

At the completion of the study, participants were able to keep the footwear as allocated (e.g., shoes alone or shoes plus a textured insole) and received a $25 Coles/Myer gift card in appreciation of their participation.

### 2.6 Feasibility measures

Qualitative and quantitative data related to feasibility of the investigations were obtained via four means; participant diaries (where children and parents/carers documented daily hours of wear, adverse events, reasons for not wearing footwear, weekly comfort ratings and perceived changes in activity levels over the 4‐week trial period measured via visual analogue scale valid for the population [21]), administration related to recruitment opportunities (number of enquiries, number of people meeting/not meeting the eligibility criteria and enrolment statistics), clinician‐assessed outcome measures (valid and reliable measures of balance [22] and endurance [23], conducted in person, at baseline prior to randomisation and at follow‐up 4 weeks post dispense) and a semi‐structured telephone interview with parents/caregivers conducted 2 weeks post‐trial completion (covering five questions, detailed below). Measures and data collection time points are in Table 1.

**TABLE 1:** Feasibility measures, data type and collection time points.

Domain	Data‐type	Participant or parent/caregiver	Measure	Method of collection	Collection timepoint
Demand	Quantitative	Parent/caregiver	1. Number of queries/eligibility of potential participants	Telephone interview	Pre‐trial
			2. Enrolment		
Acceptability	Qualitative	Parent/caregiver	Acceptability of intervention	Telephone interview questions: Were there any issues or concerns with getting your child to wear the shoes (plus or minus insole as relevant) compared to the normal experience of getting them to wear new shoes?	2 weeks post‐trial
	Qualitative	Parent/caregiver	Adverse events	Participant diary	4 weeks
	Quantitative	Participant	Comfort	Visual analogue 100 mm scale anchored with ‘most uncomfortable’ at the zero and ‘most comfortable’ 100 mm	4 weeks
Implementation	Qualitative	Parent/caregiver	Wear time	Reasons footwear was not worn during trial period	4 weeks
	Quantitative	Parent/caregiver	Wear time	Daily diary of hours of wear	4 weeks
Practicality	Qualitative	Parent/caregiver	Trial engagement	Telephone interview questions What was convenient and what was inconvenient, about participating in this study? Is there anything else you think we could do to make it easier for children/caregivers to be involved in this study? Any other concerns about participation	2 weeks post trial
Adaptation	Qualitative	Parent/caregiver	Physical activity participant	Telephone interview Thinking about your child's movements and participation in activities before getting the shoes (plus or minus insoles where relevant), do you think there has been any differences between them and what you've seen over the 4‐weeks of the trial?	2 weeks post trial
	Quantitative	Participant	Physical activity participation	Participant diary Visual analogue 10 mm scale where 0 was ‘much less than last week’, 5 equalled ‘same as last week’ and 10 was ‘much more than last week’	4 weeks
Limited efficacy	Quantitative	Participant	Balance	Balance domain and age related quartile on the movement ABC‐2 battery of fine and gross motor tests [movement ABC‐2]	Baseline 4 weeks
			Endurance	Six‐minute walk test	Baseline 4 weeks

*Both the Movement ABC‐2 balance domains and the six‐minute walk test are valid and reliable measures of balance and endurance in the population of interests' age group.

### 2.7 Process

Interested families contacted their preferred recruitment site and completed a telephone screening process, conducted by the administration support that sought their child's motor coordination status as related to the eligibility criteria. Where a motor coordination disorder was suspected but not formally diagnosed, potential participants were offered a free clinician‐based screening appointment to complete the Movement ABC‐2 gross motor assessment domains. Children assessed to be on the 15th percentile or lower for the balance domain were eligible for inclusion.

After consenting, participants attended a 1‐h baseline assessment session, where height, weight and shoe size was obtained and balance and the six‐minute walk test were assessed. Baseline assessments were conducted in the participant's own shoes.

Participants were randomised following baseline assessment (Figure 1). All participants were sized and fitted for their allocated footwear (e.g., shoes alone or shoes plus a textured insole) by experienced podiatrists (HB and CW). Participants were given verbal and written instructions on wearing the footwear. If the appropriately sized shoes or insole were not available on the day, a second appointment was made to complete the dispense of footwear. Participants were also given a diary (hard copy or electronic version), asked to wear the footwear as allocated for 4 weeks and complete the diary weekly before returning for follow‐up assessment. The diary purposely did not ask children to disclose their allocated footwear.

At 4‐weeks, the balance and six‐minute walk test measures were repeated in the footwear as allocated and diaries collected.

Two weeks following study completion, parents/caregivers participated in a 10‐min semi‐structured telephone interview, organised at a mutually convenient time. Interviews were conducted by a single author (HB), audio‐recorded with permission from parents/caregivers.

Screening, baseline and follow‐up assessments were conducted by experienced paediatric clinicians (NR and CMW/AJ), all of whom have over 15 years of experience working with children who have motor control disorders and expertise in using assessment tools. To reduce potential bias, clinicians who collected the follow‐up data (NR and AJ) remained blinded to group allocation, with participants and parents/caregivers asked to not discuss group allocation during sessions. Data from participant diaries were collectively extracted after the completion of all participants by one author (NR) blinded to the group and not involved in data analysis. Another author (HB) divided participants into allocation groups and completed the quantitative analysis. De‐identified audio recordings of the telephone interviews were transcribed by a research assistant independent of the study prior to analysis. Qualitative data were initially themed by an author not involved in data collection (JC), with a second author (CW) reviewing statements for alignment against the feasibility domains.

### 2.8 Data analysis

Descriptive statistics were used to indicate participant characteristics. Quantitative data were manually transferred from Microsoft Excel (Microsoft Corp, Redmond, Washington), into international business machines statistical package for the social sciences (SPSS) Statistics for Windows, version 27.0 (SPSS Inc) for analysis.

The a priori decision was to thematically code qualitative data against Bowen's framework for feasibility studies [12]. This deductive method of analysis allowed for the statements to be individually aligned with the domains within the framework. Statements varied in length and theming was performed even if there was one sentence aligning with the domain. JC and CW acknowledge their clinical experiences in supporting children with motor control disorders prior to this process and how this may influence the theming process [24].

Changes in self‐reported comfort, wear time and activity levels were explored using independent, two‐tailed *t*‐tests [25]. Differences between baseline and follow‐up measures were compared using a mixed between‐within analysis of variance, with partial eta squared to identify effect size change, interpreted as ≥0.01 = small effect, ≥0.06 = moderate effect, ≥0.14 = large effect [25, 26].

Quantitative outcomes associated with the feasibility domains of *Demand, Acceptability, Implementation* and *Adaptation* were considered strong if they exceeded or were within 10% of the recruitment target (*Demand*) or the mean of the shoe group outcomes (*Acceptability, Implementation, Adaptation* and *Limited efficacy*) respectively, moderate if they were within 30% of these measures and low for all other outcomes. A post hoc power analysis was performed retrospectively for dependent variables via G*Power [27]. Statistical significance was set at ≤0.05.

## 3 RESULTS

**FIGURE 2:**
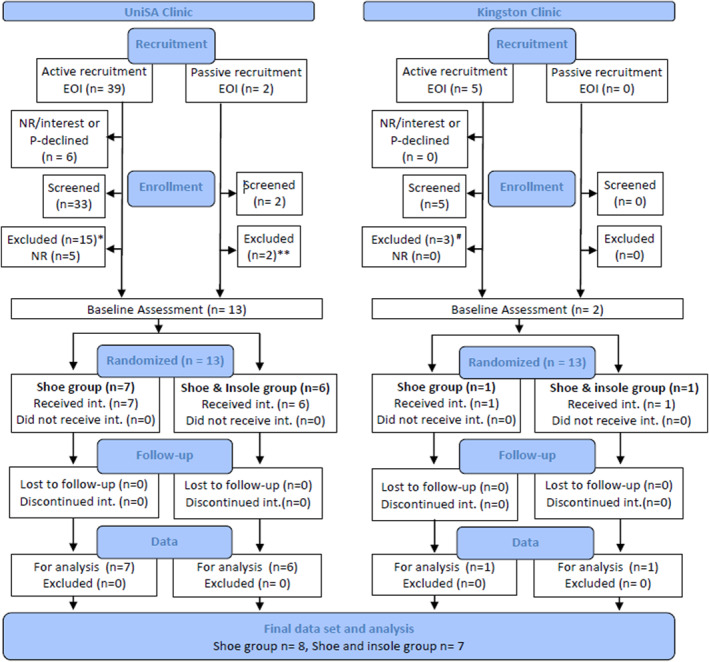
Summary of recruitment and participation. Shoes group received ASICS contend sneakers. Insole group received ASICS contend sneakers and a Naboso Activation textured insole. Passive recruitment = flyer advertising in the clinic and via social media advertising of the author networks. Active recruitment = paid Facebook^TM^ advertising. EOI—expressions of interest, Int—intervention, NR—no response, P‐declined—parent declined, UniSA—University of South Australia. * Reasons for exclusion were: child was receiving therapy services (*n* = 3), outside age range (*n* = 2), child was already wearing orthotics (*n* = 4), parent advised their child doesn't meet eligibility criteria (*n* = 1), child did not have motor coordination issues (*n* = 4), child had a broken leg (*n* = 1). ** Reasons for exclusion were: child did not have motor coordination issues (*n* = 1) # Reasons for exclusion were: Child was already wearing orthotics (*n* = 3).

A total of 15 participants were recruited between May and October 2022 (Table 2). Of those recruited, eight were randomised to the control and seven to the intervention group (Figure 2). All participants completed baseline and follow‐up measures for balance testing, one participant from the shoe group sustained an ankle injury during their baseline walk test and was therefore excluded from the associated analysis. Data regarding weight was not available for one participant within the control group, hours of use were also excluded for a further participant in the control group due to the inability to identify dates and hours of wear (Supporting Information S1).

**TABLE 2:** Participant characteristics.

	Total (*n* = 15)	Shoe group (*n* = 8)	Shoe and insole group (*n* = 7)
Male:female	10:5	6:2	4:3
Age (years), mean (SD)	8.61 (2.2)	9.20 (2.0)	8.79 (2.3)
Height (cm), mean (SD)	134.70 (16.0)	133.25 (15.3)	136.36 (16.7)
Weight (kg), mean (SD)	33.17 (13.1)^a^	33.09 (15.1)^a^	33.17 (10.5)

Abbreviation: SD, standard deviation.

^a^ Missing data from 1 participant.

### 3.1 Outcomes

#### 3.1.1 Demand

Demand was considered moderate, with 15 participants from 46 enquiries (41 at the University, 5 at the private practice) enrolled over six months (Figure 2). Importantly, all enrolled participants were recruited via the active recruitment phase (August to October), resulting from the Facebook^TM^ advertisement. Two expressions of interest were obtained prior to the advertising release, however, neither child met our inclusion criteria (Figure 2). Site comparisons noted strong *Demand* at the University (with an enrolment of 13 from a target of 15 participants (87%)) and low *Demand* at the private practice site (with an enrolment of 2 from a target of 4 participants (50%)). From those who enquired but were not eligible to enrol, the most common reasons were that the children were assessed above the 15th percentile for motor coordination (*n* = 8) or were already wearing foot orthoses (*n* = 7) (Figure 2).

#### 3.1.2 Acceptability

Wearing the footwear as allocated and the convenience of engaging in the trial appeared to be acceptable to children and parents/carers, with some notable considerations raised. Most parents (*n* = 10) reported no concerns with getting their child to wear the footwear, regardless of group allocation. A common preconception among parents of children in the insole group was that their child would struggle to wear them due to textural differences and sensory issues associated with their motor control concerns. This was not, however, reported by any parent of a child from the insole group during the post‐study telephone interviews. One parent highlighted that there was even some excitement and novelty associated with being involved in the study.

He was quite excited, he was quite comfortable wearing them, [he] had no problems, so yeah, not an issue at all

(Parent of participant 3)

Whilst many reported that their children were happy to wear the footwear, there were a few who articulated that they didn't feel that the research intervention aligned with the child's ability given their disability. Parent of participant 2 stated:

I was under the impression they were Velcro shoes and we always buy Velcro shoes for him. Because of his mobility issues, he’s not very good at motor skills so he can’t do up laces

Lace up shoes were an issue reported by several families due to their child's related motor issues. Families even expressed an element of surprise that accessible shoes with Velcro or a zipper fixture were not offered. Consequently, many reported decreased independence as children were required to ask for help from peers, parents and teachers in putting on their shoes. However, some families reported the capacity to find solutions to their perceived barriers. One family purchased Velcro straps that could replace the laces on the provided footwear. While another child (despite the laces presenting an initial barrier to independence) ‘asked to focus on learning how to tie laces with her [*occupational therapist*]’ and ‘since the study has finished, she's been continuing to favour the shoes that had the laces and the insoles” (Parent of participant 14).

Quantitative review of the child's reported comfort of the footwear identified that both groups rated the footwear as over 70% comfortable, with less than 10% difference between group means for the shoe and shoe plus insole groups observed (mean difference 4.66 mm, *p* = 0.64) (Table 3), identifying strong feasibility for insole use. Interestingly, whilst no participant recorded adverse events within the specific allocated space of the participants' diaries, foot pain or discomfort was noted as a reason to not wear the footwear by five children, four of which were in the insole group. One insole group member's recording related to the length of activity conducted on the day (‘we went shopping and walked a lot’) and another to perceived ‘hardness’ (‘it felt a little hard to walk on the soles inside the shoes’). The final three pain reports relate to ‘general’ uncomfortableness (‘first day uncomfortable on arch of foot’, ‘uncomfortable’ and ‘the shoes sting on the top of the foot’) as reported by two children from the insole group and one from the shoe group respectively). All children continued wearing the footwear and did not report any ongoing issues during or at the end of the trial.

**TABLE 3:** Comparisons of wear hours, activity change and comfort ratings between groups over 4 weeks of use.

	Shoe group	Shoe plus insole group	Feasibility rating	*p*
	Mean (SD), (*n* = 8)	Mean (SD), (*n* = 7)		
Average comfort (mm)	77.34 (24.3)	71.79 (20.3)	Strong	0.64
Total wear (hours)	175.57 (69.0)^a^	125.64 (40.3)	Moderate	0.12
Average wear per week (hours)	43.89 (17.2)^a^	31.41 (10.1)	Moderate	0.12
Activity change (mm)	13.38 (54.6)	−5.28 (52.6)	Low	0.51
Average activity change per week (mm)	3.34 (13.7)	−1.32 (13.1)	Low	0.51

*Note*: Negative numbers indicate reduced activity. Statistical significance ≤0.05. Feasibility ratings indicate difference of insole group means compared to shoe group means, calculated as percentage and rated as strong, moderate and low when within 10%, 30% or over 30% respectively.

Abbreviation: SD, standard deviation.

^a^ Missing data from 1 participant (*n* = 7).

#### 3.1.3 Implementation

A trend for the shoe group to wear their allocated footwear longer than the insole group was identified for total hours of wear over 4 weeks (mean difference 49.93 h, *p* = 0.12) and average hours of wear per week (12.48 h per week, *p* = 0.12) suggesting moderate feasibility ratings for the insoles (wear time of 28% less than shoe group), although neither outcome was statistically significant (Table 3). Overall, five ‘non‐pain/discomfort related’ themes were identified from participant diaries describing reasons as to why participants had occasions when they did not wear their footwear, including: (1) weather (e.g., hot or wet weather conditions), (2) activities requiring different footwear (e.g., dance, swimming carnival, camp, formal school uniform requirements, netball finals), (3) Indoors/home (participants not wearing shoes while at home, during recess/lunch indoors or due to being home during school holidays/weekends), (4) Illness (and staying home sick) and (5) Forgot.

#### 3.1.4 Practicality

Practicality sought to determine two outcomes; practitioner and/or service provider resource considerations and reported convenient and inconvenient aspects of engaging in this trial from the parents/carers perspective.

Practitioner/service provider resource commitment was predominantly expended in clinician time, estimated to take just under 60 h in total. Specifically, it takes up to 40 min to screen a child using the full Movement ABC‐2 full battery of gross and fine motor testing [19] and an hour per participant, per session, to collect outcome data. As 40 children were screened for inclusion (Figure 2) (27 h) and 15 participants enrolled and required two sessions of data collection (30 h), this does suggest that a larger randomised control trial (RCT) would need to factor an allowance of ∼4 h per participant in practitioner time. The administration tasks associated with enrolling participants and managing appointments were not formally monitored, however, were not reported as burdensome.

From the parents/carers perspective, the administration requirements for participation in the study were viewed as minimal and convenient.

They were flexible with times which was good so we could be seen on a day I did not work

(Parent of participant 9).

Parents particularly valued the ability to fill forms out electronically. Additionally, many parents identified access to physiotherapy/podiatry gross motor skills assessment for their child as a key benefit and overall parents were impressed with the quality of clinicians particularly around the child centredness of interactions.

It was good for us to have an assessment and see where she was at…and to engage with a professional, like a physio.

(Parent of participant 10).

I think the fact that he was spoken to as the person who was there more so than me made him feel more comfortable. I think that was really good.

(Parent of participant 7).

Some parents indicated concerns regarding the demands of the assessment protocol, particularly with the 6‐min walk test.

The other thing that was hard was the walking for 6 minutes. We made it engaging with them giving high fives etc, but they found this hard.

(Parent of participants 12 & 13).

The convenience of attending appointments was very site specific. All parents involved in the study articulated parking and distance as impactful in their experience of the study. Of the two families that had appointments at the podiatry private practice, free parking was available and seen as a critical factor in their participation in the study.

Without the parking I might have given the study a miss

(Parent of participant 15).

However, for the families attending the University, who were required to travel into the city to attend their appointments, the travel time and associated city parking proved challenging.

It was very inconvenient to come all the way into the city, it took an entire morning

(Parent of participant 2).

Parking was a pain

(Parent of participant 1).

Parking requirements at the University site provided additional cost and in some instances, additional stress for parents due to it being ‘tricky to find’ and time/effort concerns with the travel. Some parents who noted that the travel was a barrier also reframed it positively though, viewing this as an opportunity to come into and explore the city.

When we asked parents/carers if there was anything else they would like to report, interestingly of the parents interviewed 30% explicitly stated that they had an interest in research and studies.

I was happy to do the study… I love studies, like I love doing studies, I think it’s fascinating

(Parent of participant 1).

#### 3.1.5 Adaptation

While activity was predominantly recorded quantitatively via participant diaries. Parents were asked about their perception of changes in their child's movement and participation. For the most part, parents did not feel that there was a significant difference in movement quality or participation when compared with before the study, however, a few noted positive impacts:

because he knew that the shoes were somewhat special, I feel like he was a bit more willing to give physical things a go

(Parent of participant 8, shoe group).

he seems to just be a lot more steady on his feet

(Parent of participant 3, insole group).

I felt as though her movements were more fluid

(Parent of participant 14, insole group).

Quantitative analysis identified that while the shoe group reported greater increases in perceived activity change than the insole group within their participant diaries for total activity change over 4 weeks (mean difference 18.66 mm, *p* = 0.51) and on average per week (mean difference 4.67 mm, *p* = 0.51), these differences were not statistically significant (Table 3). As the difference in reported outcomes from the insole group exceeded 30% of the shoe only findings, low feasibility for the insole use for activity change was indicated (Table 3).

#### 3.1.6 Limited efficacy

Importantly, this study was underpowered for all variables.

Analysis of within‐group changes for pre and post intervention outcomes identified a significant large effect size improvement in standardised balance scores (Wilks' Lambda 0.60, *F* (1.13) = 8.78, *p* = 0.01, partial eta squared = 0.43) and balance percentile (Wilks’ Lambda 0.62, F (1.13) = 7.91, *p* = 0.02, partial eta squared = 0.38) for both groups over time (Table 4), meaning all children improved over the course of the trial. For the 14 participants included in the walk test analysis, there was no statistically significant interaction effect between group and time for distance covered (Wilks' Lambda 0.83, *F* (1.12) = 0.69, *p* = 0.15, partial eta squared = 0.17) (Table 4).

**TABLE 4:** Comparisons of change over time and difference between group outcomes for the Movement ABC‐2 balance domain (total standard scores and percentile) and the six‐minute walk test (6MWT) from baseline to following 4 weeks of use.

	Shoe group			Shoe and insole group			Change over time within groups		Differences between groups		Feasibility rating	Power
	Mean (SD), (*n* = 8)			Mean (SD), (*n* = 7)								
	Baseline	Follow up	Mean change	Baseline	Follow up	Mean change	*p*	Partial eta squared	*p*	Partial eta squared		
Standardised balance score	4.13 (2.0)	5.88 (2.4)	1.75 (2.0)	3.86 (2.3)	7.00 (3.1)	3.14 (4.1)	0.01	0.43	0.41	0.05	Strong	0.11
Balance percentile	4.28 (2.9)	13.26 (12.3)	8.99 (11.5)	4.74 (5.9)	24.86 (25.2)	20.11 (26.7)	0.02	0.38	0.30	0.08	Strong	0.18
6MWT (m)	380.75^a^ (109.0)	418.77^a^ (87.6)	38.02^a^ (59.6)	433.64 (62.4)	445.08 (100.9)	11.44 (59.9)	0.17	0.17	0.41	0.06	Low	0.08

*Note:* Statistical significance ≤0.05. Feasibility ratings indicate difference of shoe and insole group mean change compared to shoe group mean change, calculated as percentage and rated as strong, moderate and low when within 10%, 30% or over 30% respectively.

Abbreviations: 6MWT, 6‐min walk test; SD, standard deviation.

^a^ Missing data from 1 participant (*n* = 7).

**FIGURE 3:**
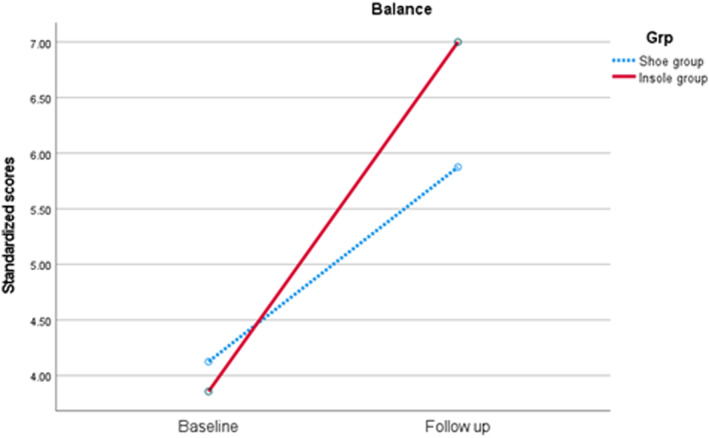
Change in Standardised balance scores between baseline and follow up (following 4 weeks of use), by group. Both groups improved over time (*p* = 0.1). No statistical differences were observed between groups.

**FIGURE 4:**
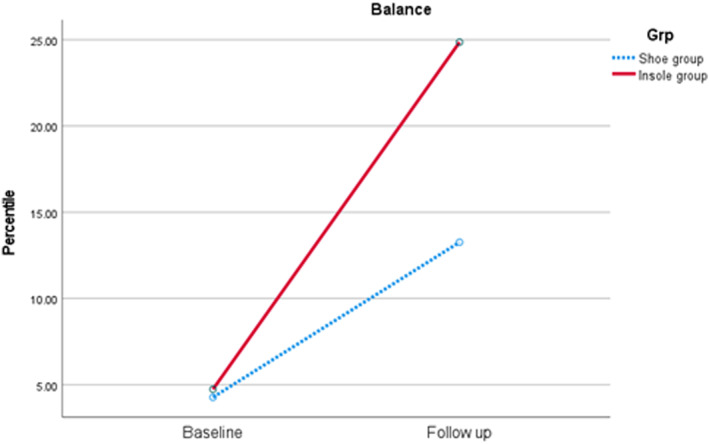
Change in Balance percentile between baseline and follow up (following 4 weeks of use), by group. Both groups improved over time (*p* = 0.2). No statistical differences were observed between groups.

**FIGURE 5:**
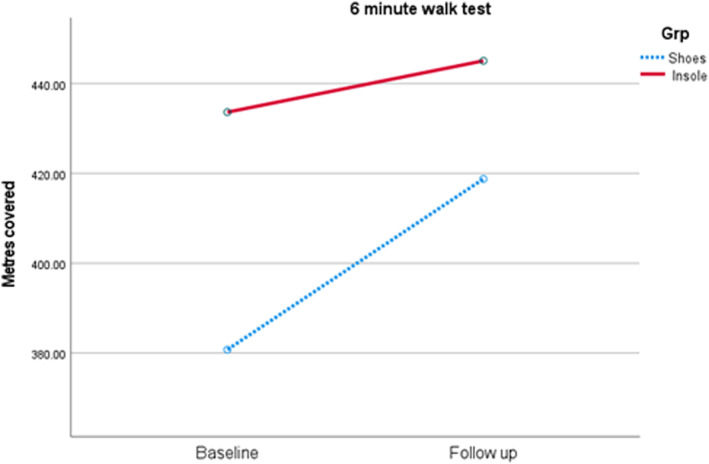
Change in Six‐minute walk test outcomes (m) between baseline and follow up (following 4 weeks of use), by group. No statistically significant differences were seen over time or between groups.

No statistical differences were identified between groups, meaning one group was not significantly improved for balance or endurance over the other (Table 4). However, a small effect size change in standardised balance scores (F (1.12) = 0.19, partial eta squared = 0.02), (Figure 3) and balance percentile (F (1.13) = 1.40, *p* = 0.29, partial eta squared = 0.10) were observed favouring the shoe and insole group (Figure 4), with a moderate effect size change in distance covered during the six‐minute walk test (F (1.12) = 0.73, *p* = 0.41, partial eta squared 0.06) favouring the shoe only group (Figure 5). The feasibility of the insole use for these measures were strong for balance impact and low for endurance change respectively (Table 4).

## 4 DISCUSSION

This is the first known study exploring the feasibility of a multi‐site randomised control trial investigating efficacy of textured insoles for children with motor coordination disorders. Our findings identified four key enablers of engagement related to *Practicality* and three key recommendations to improve *Demand, Acceptability* and *Practicality* respectively for people involved in future studies. If these enablers can be maintained and the recommendations instigated, the feasibility of a larger‐scale RCT appears supported. Given the effect of the intervention favoured improvement of balance, while the control favoured endurance respectively, it also appears prudent to identify if these seemingly simple interventions can positively impact function in children with motor coordination concerns.

All enablers of engagement reported by participants within this study related to *Practicality. Practicality* speaks to the extent to which an intervention can be delivered within the limits of time, resources and commitment of participants [12]. Within our study, this was also the only domain that included a focus on parents/carers perspectives, with outcomes reflecting their experiences. Specifically, reported enablers of engagement were gaining access to gross motor assessment reports for their child/ren, the opportunity to be involved in research, ease of parking and flexibility of appointment schedule. There were no concerns raised from the child's self‐reported outcomes for *Practicality* or any other domains that related to enabling engagement with this study.

Of the three key recommended protocol modifications identified from our findings, two are simple to employ. Firstly, offering Velcro fastenings for the shoes where required would improve children's and parent/carers *Acceptability* of the footwear. Secondly, using a two‐minute walk test [28] rather than a six‐minute walk test to reduce fatigue and injury risk in participants would positively impact *Practicality*. Interestingly, the concerns regarding fatigue resulting from the walk test protocol raised by children and parents/carers were also reported, informally, by clinicians at both sides after observing higher than expected levels of fatigue in the children after walking for 6 minutes and an injury in one child who fell during the test. The seemingly valid concern that fatigue may increase injury risk in this population justifies this recommendation being accepted. Encouragingly, employing these recommendations will not impact on identified enablers of engagement, nor alter the validity or reliability of the outcomes.

The third recommended protocol modification focuses on *Demand* and requires a more considered response. Recruitment was stronger at the University site compared to a private practice site, despite the feedback also identifying the ease of parking and increased flexibility of appointment times at the private practice as enablers. Indeed, the lack or ease of parking was the most frequently reported barrier and enabler at the respective sites. Given the timeline required to recruit 15 participants into this study (e.g., six months) multiple additional recruitment sites or large healthcare services/group allied health practices, who routinely work with children with diagnosed motor control concerns, would improve the feasibility of a larger‐scale RCT. This may also assist in reducing the time commitment for practitioners considering the diagnosis of gross motor skill concern would either already be established or the assessment of gross motor skill would form part of the new client screening process. Seeking disability‐related service providers located in urban areas with research knowledge and infrastructure, an existing large client population, and flexibility of services appears to be the best options. Recruitment would also be best supplemented with the ability to offer gross motor assessment reports, greater flexibility of appointment times and convenient parking. Considerations of the cost associated with the shoes and/or textured insoles will also need to be factored into further research in this area.

Despite these recommendations requiring a shift of sites/agencies, investing in further investigations appears warranted. Within the limits of this (underpowered) study, some individuals within the intervention group saw an ‘elevation’ of balance percentiles from ‘significant motor impairments’ (i.e., on the 5th percentile or below) to typical motor control function levels (i.e., equal or greater than the 16th percentile) [29]. Furthermore, children who were randomised to shoes alone reported (non‐statistically‐significant) improvements in activity levels and achieved better endurance measures when compared to the shoes and insole group. Limitations on statistical power, potential Hawthorne [30] and placebo [31] effects mean these trends in improvement cannot be attributed to the interventions with any confidence, however, the ease, low cost and acceptability of insoles and shoes indicates that quantifying their impact would be sensible. If those trends were to be maintained in a robust, powered RCT, the use of textured insoles would be a low cost/high value intervention for those with balance concerns, whilst also potentially adding to the evidence for the use of well fitted quality shoes for endurance and activity benefits.

Several limitations should be kept in mind when interpreting the findings of the current study. The primary concern remains that we were statistically underpowered for all variables. There are also difficulties associated with blinding in studies where a true ‘control’ group (e.g., no intervention) cannot be managed without introducing ‘demoralisation bias’ in participants when ‘they received nothing’ [32]. The protocols for each site were also nuanced in line with the availability of clinicians. For the University site, a second clinician was on hand to fit and dispense footwear as allocated, leaving the same (original) clinician to conduct baseline and follow up outcome measures whilst remaining blinded to intervention. At the private practice site, the assessing clinician was the only clinician on site, therefore dispensed the footwear as allocated and a second clinician, blinded to allocation and conducted the follow up measures. The impact the difference in protocol had on outcomes as reported is unknown.

## 5 CONCLUSION

Preliminary findings support the feasibility of conducting a larger scale RCT after implementing three key recommendations to enhance demand (partnering with a larger organisation for recruitment), acceptability (providing velcro shoes fastenings) and practicality (using a 2 min walk test). Furthermore, progressing to a larger well‐powered RCT is justified to confirm our preliminary efficacy findings that textured insoles may be a simple, low‐cost intervention to enhance children's balance, adding value to the noted benefits of well‐fitting, quality footwear for children's endurance and activity.

## AUTHOR CONTRIBUTIONS

**Helen A. Banwell**: Conceptualization; data curation; formal analysis; funding acquisition; investigation; methodology; project administration; writing – original draft. **Margarita Tsiros**: Data curation; funding acquisition; methodology; writing – review and editing. **Jessica Coventry**: Formal analysis; writing – original draft. **Narelle Ryan**: Data curation; investigation; writing – review and editing. **Cylie M. Williams**: Conceptualization; data curation; formal analysis; funding acquisition; investigation; methodology; writing – original draft.

## CONFLICT OF INTEREST STATEMENT

CMW is an Associate Editor of the Journal of Foot and Ankle Research. It is journal policy that editors are removed from the peer review and editorial decision‐making process for the papers that they have co‐authored. All other authors declare that they have no competing interests.

## ETHICS STATEMENT

Approval was given by the Human Research Ethics Committee of the University of South Australia (Approval number 204442).

## CONSENT FOR PUBLICATION

Not applicable.

## Supporting information

Supporting Information S1

Supporting Information S2

Figure S1

## Data Availability

All available data is provided within the manuscript or in Supplementary file 1.
