# Supplementary material for: Textured insoles may improve some gross motor balance measures but not endurance measures in children with motor coordination issues. A randomised controlled feasibility trial
Source: J Foot Ankle Res. 2024 Jul 1;17(3):e12036. doi: 10.1002/jfa2.12036 (PMC11633344; doi:10.1002/jfa2.12036)
Supplement: Supplementary file 1 — Supporting Information S1 [file JFA2-17-e12036-s002.docx]

We investigated the feasibility of using textured insoles and standardised sneakers, compared to standardised sneakers alone, for potential impact on balance and endurance in children with motor coordination concerns. Our findings, while not powered, suggest the insoles ‘show promise’ for balance and identified future investigations are best placed in large, multi-disciplinary child and disability-focused health services.
