# Supplementary material for: Textured insoles may improve some gross motor balance measures but not endurance measures in children with motor coordination issues. A randomised controlled feasibility trial
Source: J Foot Ankle Res. 2024 Jul 1;17(3):e12036. doi: 10.1002/jfa2.12036 (PMC11633344; doi:10.1002/jfa2.12036)
Supplement: Supplementary file 3 — Figure S1 [file JFA2-17-e12036-s003.docx]

Supplementary File 1: Participant outcomes at baseline and following 4-weeks of shoes, or shoes and insole use.

| Participant ID | Gender | Group | Baseline measures | | | Follow up after 4 weeks of use | | |
| --- | --- | --- | --- | --- | --- | --- | --- | --- |
|  |  |  | 6MWT (m) | Balance SS | Balance (%) | 6MWT (m) | Balance SS | Balance (%) |
| 1 | F | S | NR | 1 | 0.1 | NR | 6 | 9 |
| 2 | M | S | 219.2 | 5 | 5 | 336.0 | 5 | 5 |
| 3 | M | I | 436.2 | 7 | 16 | 368.0 | 4 | 2 |
| 4 | M | I | 528.0 | 6 | 9 | 536.0 | 10 | 50 |
| 5 | M | S | 432.0 | 1 | 0.1 | 464.0 | 1 | 0.1 |
| 6 | M | I | 412.4 | 1 | 0.1 | 445.5 | 5 | 5 |
| 7 | M | S | 517.2 | 5 | 5 | 520.0 | 5 | 5 |
| 8 | M | S | 368.6 | 5 | 5 | 304.0 | 6 | 9 |
| 9 | M | S | 428.4 | 5 | 5 | 464.0 | 9 | 37 |
| 10 | F | S | 448.9 | 5 | 5 | 497.9 | 8 | 25 |
| 11 | M | I | 509.6 | 1 | 0.1 | 616.0 | 11 | 63 |
| 12 | F | I | 372.3 | 3 | 1 | 320.0 | 7 | 16 |
| 13 | F | I | 402.9 | 4 | 2 | 405.4 | 3 | 1 |
| 14 | F | I | 374.0 | 5 | 5 | 424.6 | 9 | 37 |
| 15 | M | S | 251.0 | 6 | 9 | 345.5 | 7 | 16 |

NR = not recorded, S = shoe group, I = shoe and insole group, 6MWT = 6-minute walk test, Balance SS = standard score achieved of the Movement ABC-2 balance domain, Balance (%) = balance percentile achieved during the Movement ABC-2 balance domain assessment.
